# Fluoroquinolone Resistance Linked to GyrA, GyrB, and ParC Mutations in *Salmonella* *enterica* Typhimurium Isolates in Humans

**DOI:** 10.3201/eid0911.030317

**Published:** 2003-11

**Authors:** Isabelle Casin, Jacques Breuil, Jean Pierre Darchis, Claire Guelpa, Ekkehard Collatz

**Affiliations:** * Hôpital Saint-Louis, Université Paris VII, Paris, France; † INSERM E0004-LRMA, Université Paris VI, Paris, France; ‡ Centre Hospitalier Villeneuve-Saint Georges, Villeneuve-Saint Georges, France; § Centre Hospitalier de Compiègne, Compiègne, France; ¶ Hôpital du Val d’Yerres, Yerres, France

## Abstract

We report two cases of infection with clonally unrelated, high-level ciprofloxacin-resistant, β-lactamase–producing strains of *Salmonella enterica* Typhimurium. Resistance was caused by four topoisomerase mutations, in GyrA, GyrB, and ParC and increased drug efflux. Ciprofloxacin treatment failed in one case. In the second case, reduced susceptibility to third-generation cephalosporins occurred after initial treatment with these drugs and may explain the treatment failure with ceftriaxone.

Ciprofloxacin, a member of the large and widely used fluoroquinolone group of antimicrobial drugs, is considered the empirical treatment of choice of gastrointestinal infections in adults. The fluoroquinolones have been licensed for use in humans as well as in food-producing animals. This use has raised a worldwide debate on the selection of fluoroquinolone-resistant bacteria in, and the possible circulation between, the different ecosystems concerned (*1*). While resistance to these drugs occurred quickly in *Escherichia coli* and several other enterobacteriaceae, high-level resistance to ciprofloxacin (HLRC) has been found exceptionally in salmonellae (*2*–*5*). The low prevalence of salmonellae with HLRC has been ascribed to counter selection in the environment (*6*), an observation corroborated by the difficulty in selecting HLRC mutants in vitro. This type of resistance generally involves multiple mutations in the genes encoding the quinolone target enzymes, gyrase and topoisomerase IV (*2*), and mutations in regulatory systems (*marORAB* [*7*]) or *soxRS* [*8*]) or drug efflux systems (AcrAB) (*9*). We report two cases of infection caused by multiple-resistant *Salmonella enterica* Typhimurium with HLRC in which initial therapy failed and present data on the fluoroquinolone resistance mechanism.

## The Study

### Case 1

A 74-year-old man with an aorto-femoral bypass and chronic prostatitis became febrile and was treated empirically with ciprofloxacin. After 2 weeks of persistent fever, he was hospitalized. A pyelonephritis was diagnosed and endocarditis suspected. Urinalysis and blood culture both led to the isolation of a strain of *S.* Typhimurium (STmA) resistant to ciprofloxacin, amoxicillin, tetracycline, chloramphenicol, and sulfonamide. A treatment with cefotaxime was started, the fever subsided, and the patient recovered and was discharged. He did not go for follow-up consultations.

### Case 2

A 3-month-old boy (body weight 6 kg) arrived at the hospital with severe diarrhea and fever. A stool culture indicated a strain of *S*. Typhimurium (STmB1) resistant to ciprofloxacin, amoxicillin, tetracycline, trimethoprime, chloramphenicol, streptomycin, spectinomycin, and gentamicin. Treatment was initiated with ceftriaxone IV (250 mg/day) and maintained for 7 days. Signs and symptoms improved for a few days, but fever relapsed after 2 weeks. A second stool culture indicated *S*. Typhimurium strain STmB2, with the resistance phenotype of the original isolate. A second treatment with the same antimicrobial drug IV (300 mg/day) was given for 6 days, again with apparent initial success. However, fever and diarrhea reoccurred after 3 weeks (isolation of strain STmB3 from stool sample). All symptoms disappeared after oral treatment with cefpodoxime (96 mg/day/7 days). The search for carriers within the family was positive in one, with isolation of strain STmC in a 1-year-old sibling who showed no clinical signs of infection.

All STm strains were analyzed for their lysotype, pulsed-field gel electrophoretic (PFGE) pattern, and resistance phenotype and genotype, as previously described (*10*). Strain STmA was phage nonsusceptible; all STmB and STmC strains were of lysotype 12. The last two had identical PFGE profiles after digestion of genomic DNA with *Xba*I, patterns that were clearly different from that of STmA (5 of 12 fragments were in common). Polymerase chain reaction (PCR) amplification indicated β-lactamase genes in these strains of type TEM in STmA and of type TEM and OXA-1 in the STmB and STmC strains. MICs of the antimicrobial drugs shown in the Table were determined for the five clinical strains and the reference strain *S*. Typhimurium NCTC 12416 (phage type LT2), on Mueller-Hinton medium containing serially twofold diluted antimicrobial drugs. MICs of ethidium bromide and of ciprofloxacin, cefotaxime, and ceftriaxone in the presence of the efflux pump inhibitor Phe-Arg-β-naphthylamide (Sigma-Aldrich Chimie, Saint Quentin Fallavier, France) (*11*) at concentrations of 50 mg/L were also determined. All clinical strains were similarly high-level resistant to fluoroquinolones. Isolate STmA, producing the TEM β-lactamase, was more susceptible to cefotaxime and ceftriaxone than the STmB and STmC isolates producing in addition OXA-1, and the post-treatment STm strains B2 and B3 were two- to fourfold less susceptible to the third-generation cephalosporins than STmB1 (Table). In the presence of Phe-Arg-β-naphthylamide at 50 mg/L, a concentration at which no inhibition of growth was observed in the controls, MICs of ciprofloxacin were reduced fourfold and those of cefotaxime and ceftriaxone were reduced twofold. All STm isolates showed strongly reduced susceptibility to ethidium bromide (MIC > 1,000 mg/L as opposed to <100 mg/L for the reference strain).

**Table:** Antimicrobial drug susceptibilities of the *Salmonella enterica* Typhimurium isolates

Isolate	MIC (mg/L)^a^			
	NOR	CIP^b^	CTM	CTX
NCTC 12416	0.06	0.016	0.06	0.125
A	32	16	0.06	0.06
B1	32	32	0.25	0.125
B2	32	32	1	0.25
B3	32	32	1	0.25
C	32	32	0.25	0.125

^a^NOR, norfloxacin; CIP, ciprofloxacin; CTM, cefotaxime; CTX, ceftriaxone. ^b^MICs of moxifloxacin were identical to those of CIP.

To identify mutations responsible for HLRC, the quinolone resistance determining regions (QRDR) of DNA gyrase and topoisomerase IV were determined after nucleotide sequencing of the corresponding QRDR fragments which were PCR-amplified with the respective pairs of primers: 5′CTGAAGCCGGTACACCGTCG and 5′TCGGCCATCAGTTCGTGGGC for *gyrA*; 5′TTATCGATGCTGCGCGTGCC and 5′TCGCCGCTTTCAGGGCGTTC for *gyrB*; 5′CGCCTACTTAAACTACTCCA and 5′ATCAGCGTAATCGCCGCTTT for *parC*; and 5′GACC-GAGCTGTTCCTTGTGG and 5′GCGTAACTGCATCGGGTTCA for *parE*. The QRDRs of the topoisomerases of the five strains had identical mutations in GyrA (Ser83Phe and Asp87Asn) and in GyrB (Ser464Phe), while distinct mutations were observed in ParC (Glu84Lys in strain STmA and Ser80Arg in all STmB and STmC strains). The recently described quinolone resistance-conferring gene *qnr* was absent from all STm strains as tested by PCR with the corresponding specific primers (*12*). Also, nucleotide sequencing did not show any mutation in the PCR-amplified *marORA* locus.

Electrophoretic (sodium dodecyl sulfate-polyacrylamide gel electrophoresis) analysis of outer membrane proteins indicated the loss of one of them, possibly a porin (data not shown), in the post-treatment isolates STmB2 and STmB3. These changes did not influence the levels of fluoroquinolone resistance but were associated with reduced susceptibility to cefoxitin and cefotaxime (fourfold) and to ceftriaxone (twofold) (Table).

## Conclusions

The two cases reported here show that clonally unrelated HLRC strains of *S*. Typhimurium, which may cause severe infections, are present in the community. In both cases initial treatment failed, respectively, with a fluoroquinolone and a third-generation cephalosporin. In the first case, the failure may be attributable to the multiple topoisomerase mutations (possibly in conjunction with increased drug efflux), although treatment failures have been reported in cases of lower levels of resistance to ciprofloxacin (*13*). In the second case, the reason for the initial treatment failure with ceftriaxone is less obvious. Strain STmB1 had the potential to decrease its susceptibility to third-generation cephalosporins under treatment, possibly related to the loss of an outer membrane protein**.** The high-level resistance to ethidium bromide and the increased susceptibility of the strains to ciprofloxacin in the presence of Phe-Arg-β-naphthylamide were strongly suggestive of an activated drug efflux system, such as AcrAB, the substrates of which also include cephalosporins with lipophilic side chains (*2*,*14*). The growth rates of the strains were the same as that of the reference strain, an observation which is at variance with that made with experimental fluoroquinolone-resistant mutants selected in animals (*6*) and which might imply the existence of additional compensatory mutants in the human STm isolates.

Low-level ciprofloxacin resistance in salmonellae has been observed occasionally in the environment in France (*15*) and elsewhere (*16*). Since we did not have a pretreatment isolate at our disposal, we are not certain whether any of the mutations resulting in the high-level fluoroquinolone resistance observed in strain STmA were selected under treatment. On the other hand, HLCR strain STmB may have been acquired as such from the environment or through the food chain, since neither the patient nor his sibling had ever been treated with fluoroquinolones, although no salmonellae with this phenotype have been observed to date from environmental or animal sources in France. In fact, to our knowledge, quadruple mutations affecting three topoisomerase subunits have never been reported before in salmonellae.

The unwelcome occurrence of fluoroquinolone-resistant salmonellae accumulating multiple mechanisms of resistance could compromise standard therapy of infections attributable to such pathogens. For children, an initial high-dose regimen with commonly used third-generation cephalosporins, or with oral cephalosporins, may therefore be indicated for the treatment of infections caused by *S.* Typhimurium strains such as STmB1.
